# Frequent detection of parental consanguinity in children with developmental disorders by a combined CGH and SNP microarray

**DOI:** 10.1186/1755-8166-6-38

**Published:** 2013-09-20

**Authors:** Yao-Shan Fan, Xiaomei Ouyang, Jinghong Peng, Stephanie Sacharow, Mustafa Tekin, Deborah Barbouth, Olaf Bodamer, Roman Yusupov, Christina Navarrete, Ana H Heller, Sérgio DJ Pena

**Affiliations:** 1 Department of Pathology and Mailman Center for Child Development, Room 7050, University of Miami Miller School of Medicine, 1601 NW 12th Avenue, Miami, FL 33136, USA; 2 Department of Human Genetics, University of Miami Miller School of Medicine, Miami, FL, USA; 3 Memorial Regional Hospitals, Miami, FL, USA; 4 Jackson Memorial Hospital, Hollywood, FL, USA; 5 Núcleo de Genética Médica, Belo Horizonte, Belo Horizonte, MG, Brazil

**Keywords:** Chromosome microarray, Consanguinity, Developmental disabilities

## Abstract

**Background:**

Genomic microarrays have been used as the first-tier cytogenetic diagnostic test for patients with developmental delay/intellectual disability, autism spectrum disorders and/or multiple congenital anomalies. The use of SNP arrays has revealed regions of homozygosity in the genome which can lead to identification of uniparental disomy and parental consanguinity in addition to copy number variations. Consanguinity is associated with an increased risk of birth defects and autosomal recessive disorders. However, the frequency of parental consanguinity in children with developmental disabilities is unknown, and consanguineous couples may not be identified during doctor’s visit or genetic counseling without microarray.

**Results:**

We studied 607 proband pediatric patients referred for developmental disorders using a 4 × 180 K array containing both CGH and SNP probes. Using 720, 360, 180, and 90 Mb as the expected sizes of homozygosity for an estimated coefficient of inbreeding (F) 1/4, 1/8, 1/16, 1/32, parental consanguinity was detected in 21cases (3.46%).

**Conclusion:**

Parental consanguinity is not uncommon in children with developmental problems in our study population, and can be identified by use of a combined CGH and SNP chromosome microarray. Identification of parental consanguinity in such cases can be important for further diagnostic testing.

## Background

Studies of the human genome using genomic arrays (also called chromosomal microarrays) in the last decade have revolutionized clinical cytogenetics and changed the standard of care in medical genetics [1-4]. The resolution of the studies has evolved from the megabase level of the bacterial artificial chromosome (BAC) arrays during the early 2000s to the kilobase or exon level of the current oligonucleotide arrays. The traditional microarrays were designed for detection of gene copy number variations (CNVs) by comparative genomic hybridization (CGH), and were popularly called CGH arrays. The second type of arrays containing a high density of probes for single nucleotide polymorphisms (SNPs), so called SNP array, reveals both CNVs and homozygosity in the genome. The third type of genomic arrays uses a combination of probes designed to detect CNVs and probes to genotype SNPs. The use of genomic microarrays has significantly improved accuracy and diagnostic yield in comparison with the conventional karyotype. Recently, genomic microarrays have been used as the first-tier cytogenetic diagnostic test for patients with developmental delay/intellectual disability, autism spectrum disorders and/or multiple congenital anomalies [2-4]. Many studies have shown the advantages of genomic arrays in detecting pathogenic CNVs. Our laboratory has performed CGH array studies on about 2500 patients with developmental disorders since 2005, and reported a 14% detection rate for pathogenic CNVs [5,6].

The use of SNP arrays has revealed regions of homozygosity (ROH) in the genome which can lead to identification of uniparental disomy (UPD) and parental consanguinity in addition to CNVs [7,8]. It is well known that consanguinity is associated with an increased risk of congenital anomalies and autosomal recessive diseases [9-12]. The recently published Geneva International Consanguinity Workshop report discussed the health impact of consanguinity and highlighted the importance of evidence-based counseling recommendations for consanguineous marriage and for undertaking genomic and social research in defining the various influences and outcomes of consanguinity [12]. However, the frequency of parental consanguinity in children with developmental disabilities is unknown, and consanguineous couples may not be identified during doctor’s visit or genetic counseling without microarray study as our data have shown. Here we report our study results on patients referred for developmental problems using a combined CGH/SNP array with a focus on detection of parental consanguinity.

## Results

A total of 607 proband pediatric patients were tested over a one year period, including 130 from Brazil and 477 from south Florida. Pathogenic CNVs were detected in 97 cases (15.98%). SNP genotyping suggested presence of UPD in three cases (0.49%), including two UPD20 and a UPD14. Our results suggested presence of parental consanguinity in 21 cases (3.46%) including 4 (3.07%) from Brazil and 17 (3.56%) referred from hospitals in south Florida (Table 1).

**Table 1:** Summary of array study results

**Sample sources**	**No. of proband patients**	**No. of cases with pathogenic CNV (%)**	**No. of cases with UPD (%)**	**No. of cases with ROH ≥68 Mb (%)**
South FL	477	75 (15.72)	3 (0.63)	17 (3.56)
Brazil	130	22 (16.92)	0	4 (3.08)
Total	607	97 (15.98)	3 (0.49)	21 (3.46)

**Figure 1:**
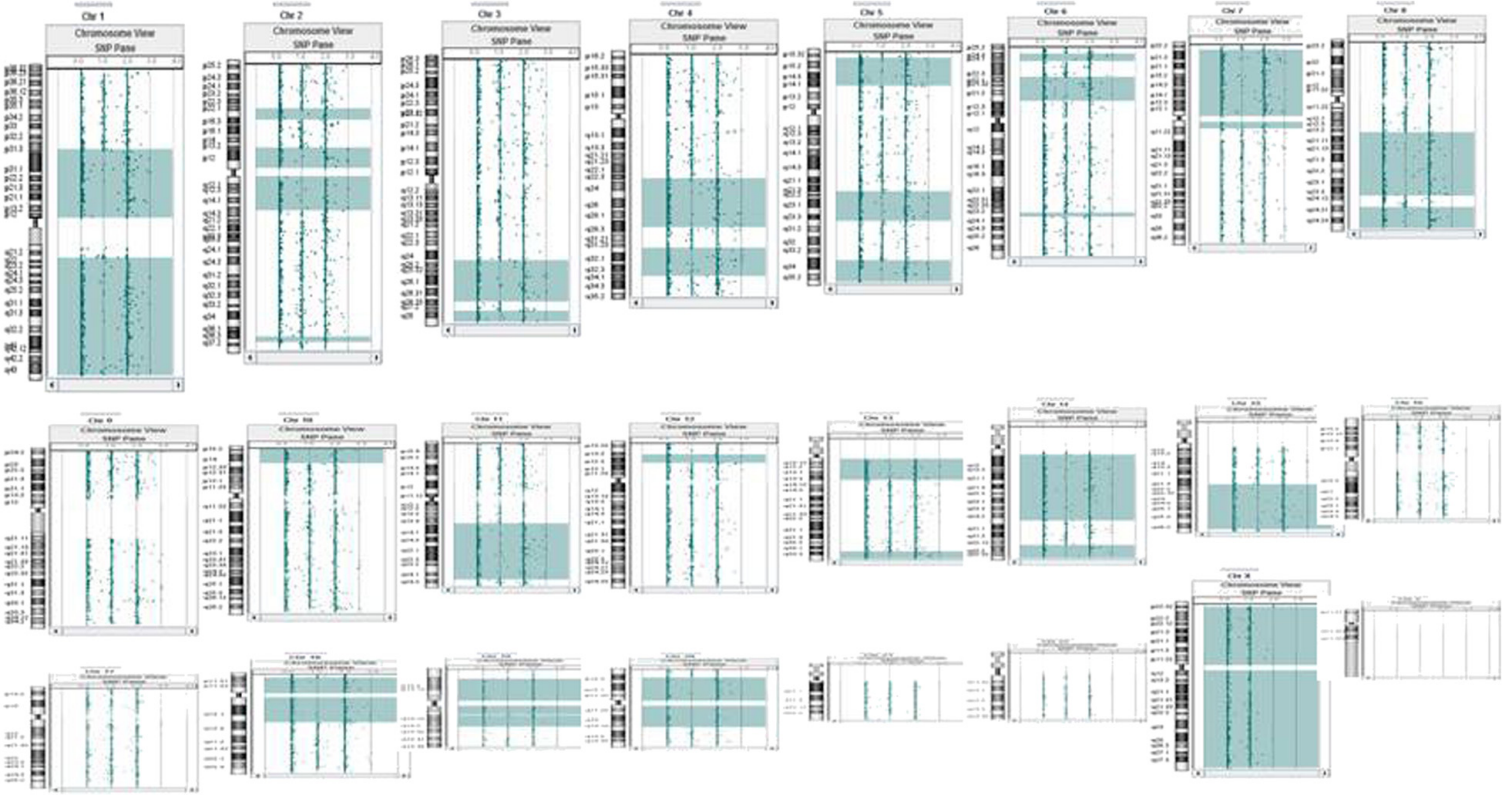
**A combined CGH and SNP array on a 6-year old girl who was initially referred for mental problems showed 598 Mb of homozygous autosomal genome (light green blocks).** This finding led to uncover the patient being conceived as the product of a brother-sister mating (first degree relatives with a coefficient of inbreeding ¼). After further studies, she had a tentative diagnosis of 2-ketoglutarate dehydrogenase deficiency, a rare autosomal recessive disease.

ROHs larger than 5 Mb were observed in 86 cases (data details not provided in this paper) including 21 with results suggesting presence of parental consanguinity. Among the 21 patients (Table 2), three had an average of 686 Mb of ROH with an estimated coefficient of inbreeding (F) ¼ (Figure 1); five had an average 329 Mb of ROH with an estimated F 1/8; five had an average of 196 Mb of ROH with an estimated F 1/16; and eight had an average 91 Mb of ROH with an estimated F 1/32. Post-testing information on parental relationship was available in 14 of the 21 cases. A parental relationship of 1st cousins was confirmed in all the 4 cases with an estimated F1/16. Among the five cases with an estimated F 1/32 suggestive of a mating type of half first cousins or first cousins once removed, three were said having parents as 2nd cousins; One couple was consanguineous but relationship was not specified; In the remaining case, parents said they are not related but from the same small city. In the three cases with F1/8 suggestive of a 2nd degree relationship, one was an uncle-niece mating; one said to be first cousins; and the other was said to be distant relatives. Among the two children with F1/4, one was a product of a brother-sister mating, and the other’s parents said they were not related.

**Table 2:** The cases with parental consanguinity

Case #*	**Age**	**Sex**	**Referring reasons**	**Size of HZ (Mb)**	**Estimated coefficient of inbreeding (F)**	**Post-testing information on mating type**
001	6 y	F	ID	598	1/4	Brother-sister
002	3 y	M	DD, DE, DF,RA	839	1/4	No related
003	18 y	M	DD, ID	624	1/4	NA
004	23 d	F	CA	345	1/8	Uncle-niece
005	7 y	M	DD	383	1/8	1st cousins
006	3 d	M	CA	285	1/8	NA
007	9 d	M	SZ	338	1/8	Distantly related
008	12	F	DD, DF	292	1/8	NA
009	12 y	F	SKS, MD	187	1/16	1st cousins
010	9 y	M	SS	196	1/16	1st cousins
011	1 y	M	DD	250	1/16	1st cousins
012	7 y	M	DF, CA, RF	208	1/16	1st cousins
013	3 y	M	DD	139	1/16	NA
014	7 y	F	DD, DF, FT	114	1/32	2nd cousins
015	2 y	F	DD, FT	85	1/32	From the same small town
016	5 y	M	DD	120	1/32	Consanguineous
017	1 m	F	CA	69	1/32	2nd cousins
018	1 y	F	ID, GR, SS	88	1/32	NA
019	6 y	F	DD	86	1/32	2nd cousins
020	18 d	M	DF, MM	72	1/32	NA
021	3 y	F	DD	96	1/32	NA

*Abbreviations*: *CA* congenital anomalies, *DD* developmental delay/disorder, *DE* diaphragmatic eventration, *DF* dysmorphic features, *FT* failure to thrive, *GR* growth retardation, *ID* intellectual disability, *MD* motor delay, *MM* multiple malformations, *NA* no information available, *RA* renal anomalies, *RF* renal failure, *SKS* scoliosis and kyphoscoliosis, *SS* short stature, *SZ* seizures. *Numbers are used for this publication only, not the real numbers used for patient management.

## Discussion

In addition to pathogenic CNVs and UPDs, approximately 3.5% of children with intellectual and/or developmental disabilities in our study populations showed greater than 68 Mb of ROH, suggesting presence of parental consanguinity. Post-testing surveys confirmed the 1st cousin relationships for all the cases with an estimated F 1/16 and presence of consanguinity in 4 out of 5 cases with an estimated F1/32 although the information from medical records was incomplete or inconsistent with the suggested mating type. In our practice, we report results suggesting presence of parental consanguinity but not specify the biological relationship of the parents as recommended by the ACMG guidelines [13], because SNP array analysis is not designed to be a paternity test or to assign a specific relationship between the parents of the proband patient. Also, the observed size of ROH may not precisely predict the true biological relationship due to variables such as recombination during meiosis, multiple loops of consanguinity or multiple generations of breeding within a relatively closed community. For the patients showing an estimated F ¼ or 1/8, we have included the percentage of homozygosity in our clinical report and stated that the result may suggest a first- or second-degree parental relationship or incestuous mating.

Incestuous parental relationships identified by SNP-based microarrays have been reported previously. One was a 3-year-old boy with multiple medical conditions showing 668 Mb of genomic homozygosity which was consistent with the patient being conceived as the product of a mating between first-degree relatives [14]. In a report on a SNP data, ROH of greater than 20% of the genome were identified in two of 5000 samples analyzed in a cytogenetics laboratory in Australia [8]. Our study has shown that a level of ROH greater than 20% correlates with an estimated coefficient of inbreeding F1/4 which is suggestive of a first degree relationship such as parent–child or brother-sister mating, as exemplified in case 001. However, an incestuous relationship, which represents a complicated social and legal issue, may not be readily identified during a doctor’s visit or genetic counseling, as shown in our post-testing survey. Genetic counseling for consanguinity can be complicated particularly when incestuous mating is uncovered by use of microarrays.

The frequency of consanguineous mating may vary significantly in different geographic regions or in different ethnic populations [12,15-17]. Our study patients were referred from hospitals in South Florida (mainly Miami-Dade and Broward counties) and Brazil. Hispanic or Latino Americans account for 65% of the population in Miami-Dade and 25% of the population in Broward (2010 census data). The Hispanic or Latino Americans in Florida originate mainly from Cuba (32%) and Puerto Rico (28%) and Mexico (9%). It was estimated in 2008 that over one million of Brazilians live in USA and about 300,000 of them live in Florida. First cousin marriage is legal in Florida. The rate of consanguineous marriage in Miami-Dade and Broward counties is unknown but is likely to be higher than the generally estimated average (<1%) in USA. The rate of consanguineous mating in Brazil was estimated as 1.6% with a heterogeneous geographic distribution and half of the consanguineous marriages have a coefficient of inbreeding F 1/16 or higher [18]. There was a consensus that consanguineous marriages are associated with an increased risk of congenital malformations and autosomal recessive disease [12]. The estimated excess risks of morbidity and precocious mortality for the children with F1/4, 1/8, 1/16 and 1/32 are about 40%, 20%, 10%, 5%, respectively [9,10]. A recent review suggests that the risk for congenital heart disease is increased in consanguineous marriages [19].

Different from previous clinical studies on the families with a history of consanguineous mating, we detected parental consanguinity in about 3.5% of children referred for developmental problems. Most of these children were referred for congenital anomalies or multiple malformations, developmental delay or intellectual disability, dysmorphic features, failure to thrive/growth retardation or short stature. The finding of parental consanguinity in these cases is highly suggestive of an underlying recessive cause. For example, following the array study, case 012 was diagnosed with Schimke immune-osseous dysplasia, an autosomal-recessive pleiotropic disorder caused by loss of function mutations in *SMARCAL1* leading to spondyloepiphyseal dysplasia, renal dysfunction and T-cell immunodeficiency [20,21]. A diagnosis of autosomal recessive spinal muscular atrophy has been suspected in case 009. In case 001, the 6 year-old girl initially referred for mental problems had a history of proximal renal tubular acidosis (RTA) at 18 months of age, basal ganglia and corneal calcifications secondary to RTA. Further diagnostic work-up has revealed increased excretion of Krebs cycle intermediate and a tentative diagnosis of 2-ketoglutarate dehydrogenase deficiency, a very rare metabolic disorder [22-24]. Involvement of multiple recessive mutant genes or a complex genetic mechanism is possible in some patients, particularly in the children as products of incestuous mating. Consanguinity may not be noted in many cases without microarray studies and identification of the biological relationship of the parents in such cases is clinically important for additional diagnostic testing. Further studies of phenotype and possible correlating metabolic deficiency, as well as sequencing of the ROH regions would determine the genetic mechanisms of the developmental problems in these children.

## Methods

### Patients

Patients, all pediatric but one, included in this report were referred for developmental delay or intellectual disability, autism spectrum disorders, and/or congenital anomalies from November 1, 2011 to March 5, 2013. Approximately 80% of the patient samples originated in the Genetics Clinic at the University Miami and other hospitals in south Florida. However, many of the patients were of Latin American or Caribbean descent or referred from Latin American countries. The remaining 20% of patient samples were received from Brazil.

### Microarray studies

A combined CGH and SNP array, Agilent SurePrint G3 4 × 180 K (Agilent, Santa Clara, USA) has been used in our laboratory as a clinical test for over a year. The array contains 180,000 oligonucleotide probes, including approximately 120,000 CGH probes and 60,000 SNP probes with a resolution of 5 ~ 10 Mb for ROH detection. Array studies were performed using a protocol recommended by Agilent. Briefly, DNA was prepared from a blood sample and purified using the QIAamp DNA Blood Midi Kit (Qiagen) or DNeasy Blood & Tissue Kit (Qiagen) when a sample was less than 0.5 ml. DNA was quantified, digested and then labeled with Cy5 (testing DNA) and Cy3 (reference DNA) and hybridized to the microarray at 65°C for 21 ~ 24 hours. The microarray was washed and analyzed using an Agilent C microarray scanner and CytoGenomics Edition 2.0.6.0 software.

### Criteria for reporting

We used the same criteria for detection and interpretation of CNVs as previously reported [5,6]. A size of 20 Mb of interstitial ROH or 10 Mb of telomeric ROH was used as the threshold for UPD when ROH was observed on a single chromosome [7,8]. For detection of parental consanguinity, we calculated the theoretic size of ROH for coefficient of inbreeding (F) 1/4, 1/8, 1/16, and 1/32 respectively using the total 2,881 Mb of autosomal genome (GRCh37/hg19) and its variable range using the middle line between theoretic average sizes (Table 3). Based on this calculation, a result was reported suggesting presence of parental consanguinity when ROHs were observed on multiple chromosomes with a total size ≥68 Mb which correlates with an estimated F 1/32. However, we did not specify the estimated coefficient of inbreeding and the likelihood of biologic relationship of the parents in our laboratory report. ROHs on multiple chromosomes with a total size of 10 ~ 67 Mb or ROHs on a single chromosome which do not meet the criteria for UPD were reported as identical by decent (shared parental ancestry). We reported the individual ROH with a size ≥5 Mb either on a single chromosome or on multiple chromosomes to clinicians (data not shown) because rarely an ROH may contain recessive mutations responsible for the clinical phenotype. We have reported and interpreted ROH using above described criteria starting in late 2011 and our practice appears to be consistent with the ACMG standards and guidelines recently published [13].

**Table 3:** Theoretic size of ROH for coefficient of inbreeding (F) expected from the total 2881 Mb autosomal genome (GRCh37/hg19)

**Mating type**	**Degree of relationship**	**Coefficient of inbreeding (F)**	**Theoretic proportion of identical by decent (%)**	**Expected ROH size and range (Mb)**
Parent–child/brother-sister	1st	1/4	25	720 (540 ~ 1080)
Uncle-niece/aunt-nephew/double half cousins	2nd	1/8	12.5	360 (270 ~ 539)
First cousins/half uncle-niece	3rd	1/16	6.25	180 (135 ~ 269)
Half first cousins/first cousins once removed	4th	1/32	3.125	90 (68 ~ 134)

### Retrospective survey on parental consanguinity

Referring clinicians were contacted to obtain information on the parent’s family relationship of the children for whom laboratory results suggested presence of parental consanguinity. The clinician provided information based on either the notes of clinical geneticists or family history in medical records. In some cases, we obtained information on patient diagnosis after microarray study.

This report is a summary of clinical cases tested in a CLIA certified and Florida State licensed clinical laboratory with patients identification removed. This is not an experimental research using human material and therefore no IRB approval is necessary.

## Consent

Written informed consent was obtained from the patient’s guardian/parent/next of kin for the publication of this report and any accompanying images.

## Abbreviations

ACMG: American college of medical genetics; BAC: Bacterial artificial chromosome; CGH: Comparative genomic hybridization; CNV: Copy number variation; F: Coefficient of inbreeding; SNP: Single nucleotide polymorphisms; ROH: Regions of homozygosity; UPD: Uniparental disomy.

## Competing interests

The authors declare that they have no competing interests.

## Authors’ contributions

YSF analyzed the laboratory data, drafted and finalized the manuscript. XO and JP performed microarray analysis and prepared the laboratory data. SS, MT, DB, OB, RY, CN, AHH, SDJP clinically examined the patients and collected clinical data. All authors read and approved the final manuscript.
